# Plasmablastic Myeloma: An Unusual Cause of Peripheral Facial Paralysis

**DOI:** 10.7759/cureus.53998

**Published:** 2024-02-11

**Authors:** João Dias, Irene Pinto, Catarina Vasconcelos, Vilma Marques

**Affiliations:** 1 Physical Medicine and Rehabilitation, Centro Hospitalar de Trás-os-Montes e Alto Douro, Vila Real, PRT

**Keywords:** bell's palsy, anaplastic myeloma, peripheral neuropathy, plasmablastic myeloma, peripheral facial paralisys

## Abstract

Peripheral facial paralysis refers to the involvement of the facial nerve (VII cranial nerve) at any point along its path, which starts from its nucleus, located in the pons, and extends to its most distal branches. The etiology is heterogeneous, including viral infections, bacterial infections, trauma, and neoplasms, among others. However, in the majority of cases, the cause is idiopathic, commonly referred to as Bell's palsy. The diagnosis is therefore one of exclusion, based in particular on the physical examination. Naturally, the diagnosis is decisive in directing the therapeutic approach. However, the signs/symptoms of the various primary pathological processes can appear late in the course of the disease. This is why the Physical Medicine and Rehabilitation specialist is particularly important, since, in addition to the initial assessment, he or she monitors the patient more closely and over a longer period of time, together with the team of therapists. New clinical findings and diagnostic tests requested accordingly can dramatically change the initial diagnosis and guide new treatments. We present the clinical case of a 60-year-old man initially diagnosed with Bell's palsy, whose poor clinical evolution and new clinical findings during the rehabilitation program led to the diagnosis of plasmablastic myeloma and a radically different therapeutic approach.

## Introduction

Peripheral facial nerve palsy refers to lower motor neuron lesion of the facial nerve and can occur as a result of various medical conditions [1].

The facial nerve, also known as the seventh cranial nerve, is a mixed nerve, that performs both motor and sensory functions [2]. The motor fibers innervate the muscles of facial expression, stylohyoid muscle, posterior belly of the digastric, and the stapes. The sensory component consists of fibers that make up the nervus intermedius of Wrisberg (two-thirds of the anterior tongue). It also has a parasympathetic component with innervation of the submandibular and sublingual salivary glands and lacrimal glands [3].

Peripheral facial paralysis is characterized by a unilateral decrease in facial muscle strength, involving both layers of the hemiface: reduced/absent wrinkles in the frontal region, difficulty/incapacity to raise the eyebrow [4], lagophthalmos, Legendre's sign, Mingazzini's sign, Bell's sign, asymmetry of the labial commissure, and effacement of the nasolabial fold. Additionally, other symptoms may arise, such as xerophthalmia, hyperacusis, and taste alteration [5].

The etiology of this neuropathy is heterogeneous [6]. The facial nerve is the most susceptible to damage among cranial nerves owing to its long intracranial anatomical path and superficial extracranial location [2]. Among all peripheral facial nerve palsies, idiopathic or Bell’s palsy constitutes the majority (60-75%) of cases. Apart from Bell's palsy, there are heterogeneous but specific etiologies that cause symptomatic peripheral facial palsy, including infections such as Ramsay-Hunt syndrome or Lyme neuroborreliosis, neoplastic or otogenic lesions, autoimmune diseases, and trauma. As idiopathic peripheral facial palsy is a diagnosis made by excluding other possible causes, it is crucial to eliminate treatable factors associated with the condition before arriving at the diagnosis of Bell's palsy [6].

In order to enhance function, physical therapy is an essential part of the treatment for facial nerve injury, in addition to medication and surgery. Physical therapies are used to improve face function, speed up the healing process, and lower the risk of problems. These therapies include exercise, biofeedback, laser treatment, electrotherapy, massage, and thermotherapy [2].

## Case presentation

A 60-year-old male with a history of essential hypertension, smoking, chronic alcohol consumption, and arterial ulcer in the right lower limb presented to the local hospital with sudden left peripheral facial paralysis, preceded by ipsilateral otalgia the day before. He was evaluated by the Neurology department in the Emergency Service and denied hearing changes and sensory and motor alterations in the left hemibody. He also denied constitutional symptoms such as fever, weight loss, or night sweats. On physical examination, he displayed deviation of the labial commissure to the right, erasing of the nasolabial fold and wrinkles in the ipsilateral frontal region, and positive signs of Bell, Mingazzini, and Legendre (House-Brackmann Grade VI).

A hemogram and serum biochemistry were requested, which showed no significant alterations. A cranioencephalic CT scan showed no significant findings. The diagnosis of Bell's palsy was thus established. He was discharged from the hospital on the same day and referred to an outpatient Physical Medicine and Rehabilitation department with a prescription for acyclovir and corticosteroid therapy (with a weaning program) and an indication for eye protection measures: lubrication, sunglasses, and nocturnal occlusion.

Three days later, he was observed at the Physical Medicine and Rehabilitation outpatient department. A daily rehabilitation programme was prescribed. Physiotherapy consisted of neuromuscular re-education, proprioceptive neuromuscular facilitation techniques, cryotherapy and massage therapy.

**Figure 1:**
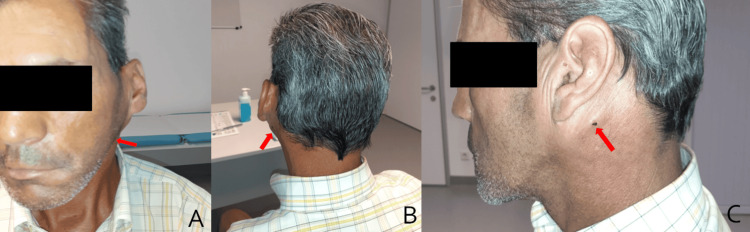
Revaluation during the rehabilitation program. Left retroauricular swelling and icteric skin.

After 15 physiotherapy sessions (approximately one month after the first observation), he was re-evaluated. There was no clinical improvement and he also presented asthenia, anorexia, polyarthralgia, and weight loss (not quantified), which had been progressing for 10 days. Physical examination revealed left retroauricular swelling, painful on palpation, left supraclavicular swelling, and jaundice of the sclera (Figure 1). He was referred to the Otorhinolaryngology department where he was hospitalised for diagnostic studies and symptomatic treatment.

**Figure 2:**
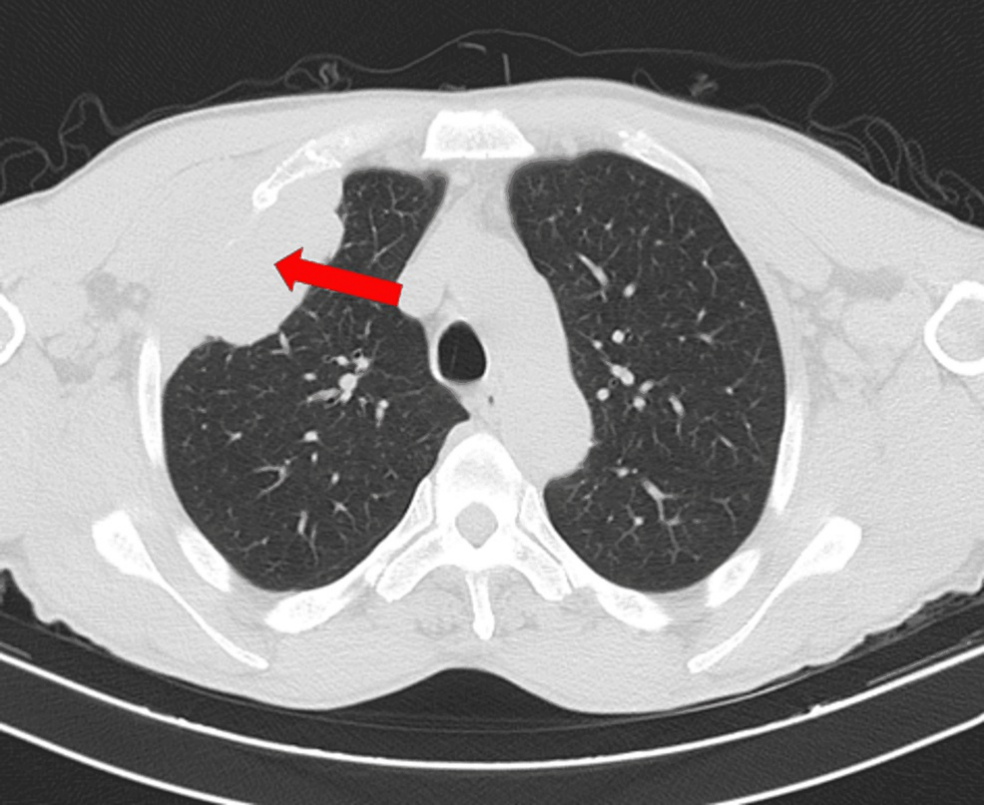
Thoracic CT scan. Right infra-clavicular solid mass, measuring 8 cm, involving and invading the second rib.

**Figure 3:**
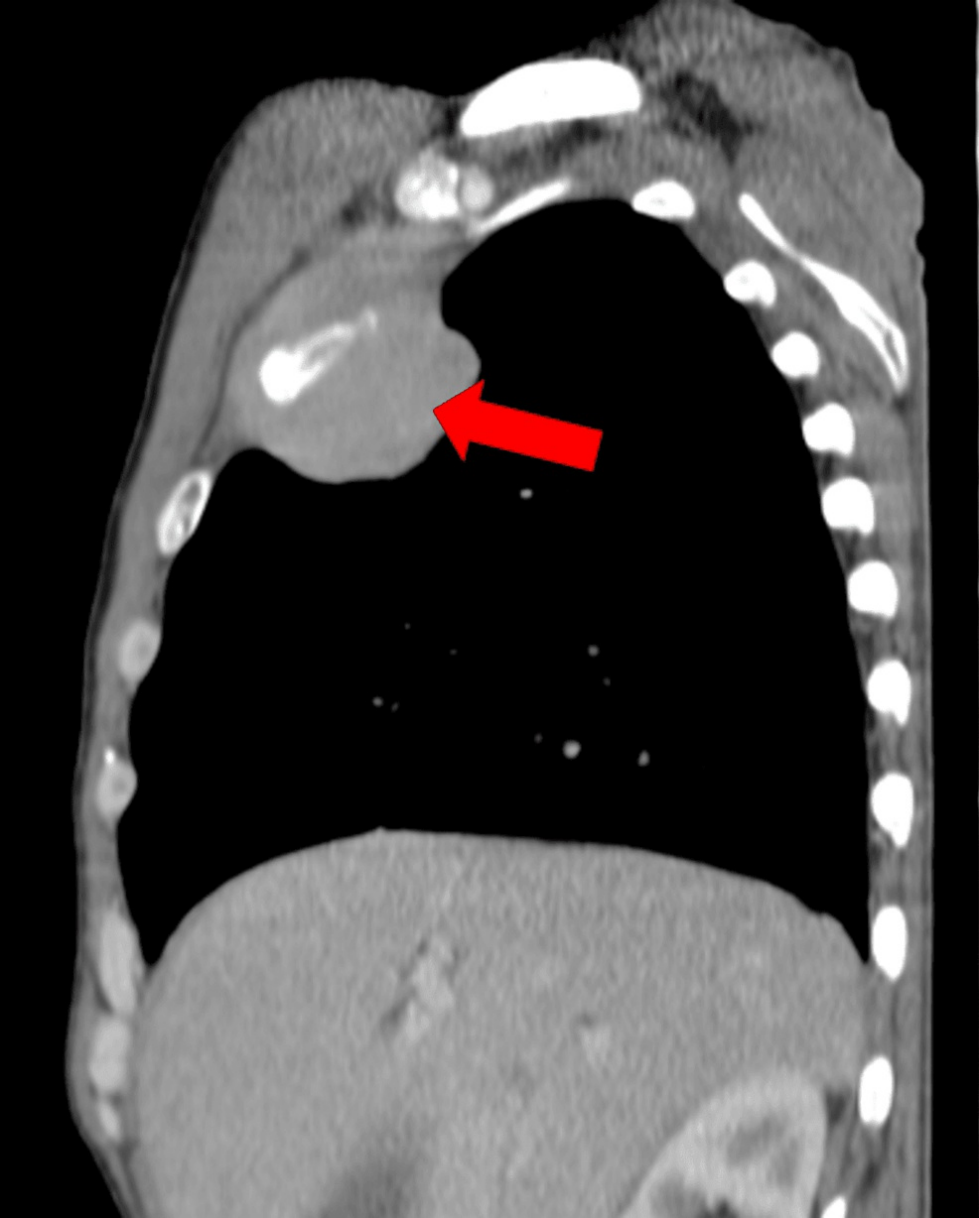
Thoracic CT scan. A 2.7 cm solid mass just to the right of the sternum.

**Figure 4:**
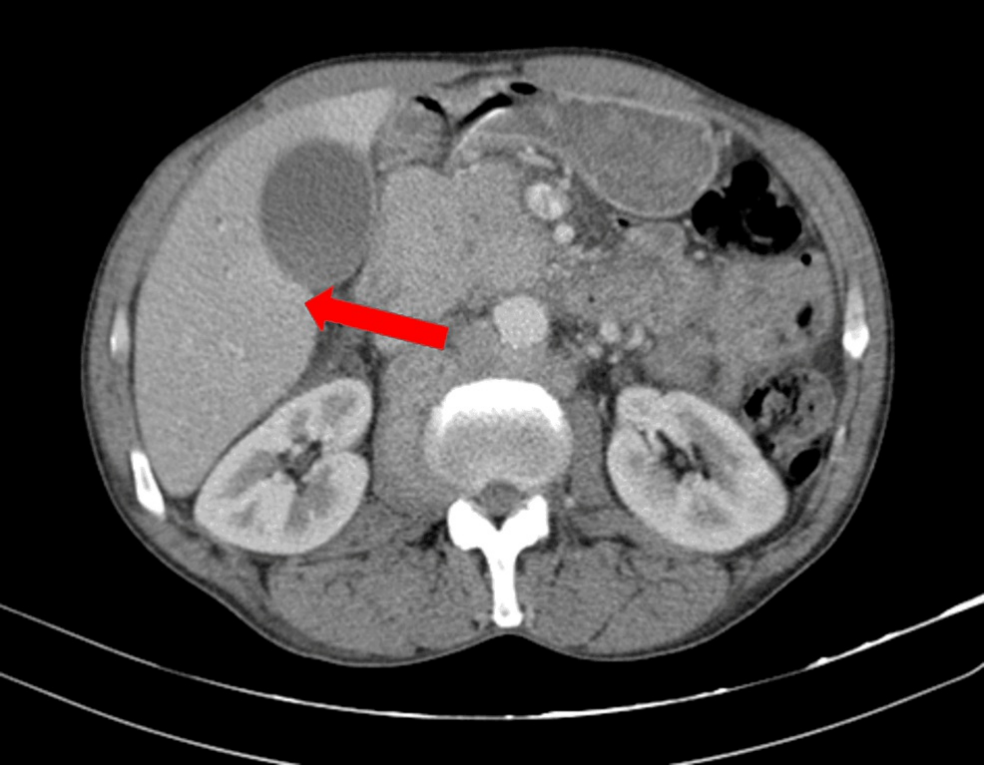
Abdominal CT scan. Liver and pancreas globose, with preserved structure. The pancreas exerts a compressive effect on the hepatic hilum and causes dilation of the bile ducts.

On admission, he had macrocytic anemia (11.50 g/dL), hyponatremia, hypoalbuminemia, and altered liver function tests. A chest X-ray showed a 6.3 cm oval opacity in the transition from the middle to the upper third on the right. CT scan (Figures 2-4) showed multiple space-occupying lesions with invasion of bone structures (retroauricular, supraclavicular, sternal), a periaortic adenopathic conglomerate, a suspected infiltrative lesion of the iliac bone, and a globose liver and pancreas leading to obstructive jaundice.

**Figure 5:**
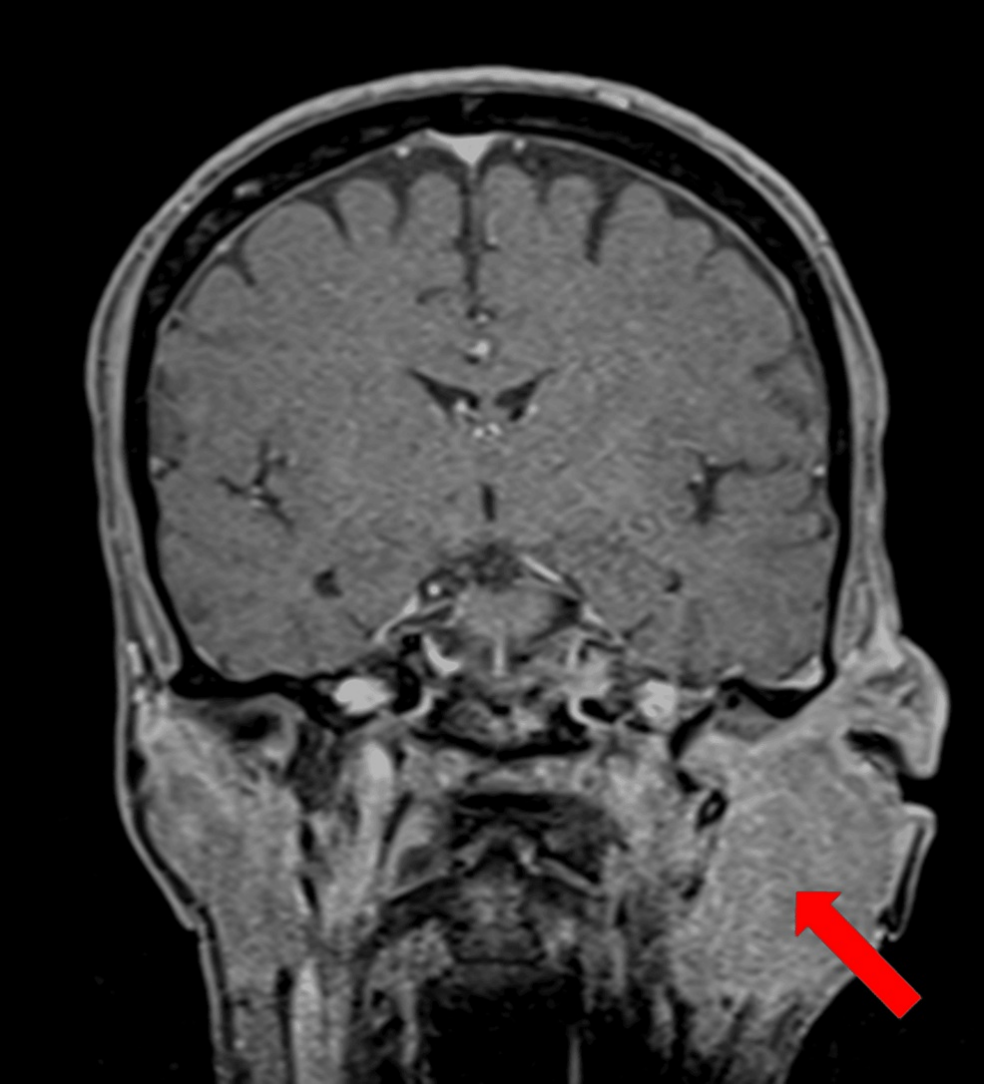
Head MRI. Bulky infiltrative formation with expansion into the parotid gland.

**Figure 6:**
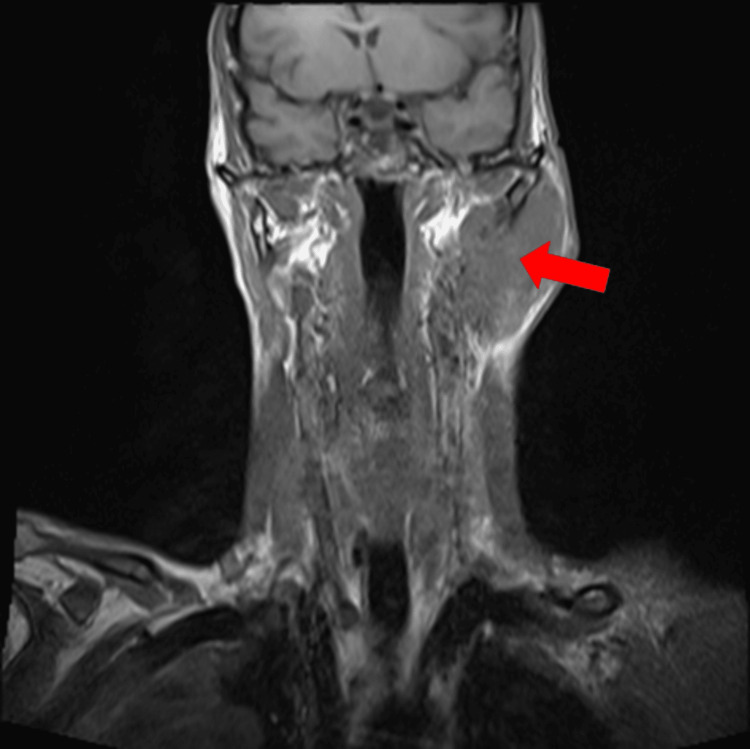
Head and neck MRI. A voluminous formation, apparently solid and vascularized, with an infiltrative and relatively non-destructive character, with great expansion of the parotid gland, the neck muscles, especially the sternocleidomastoid.

MRI of the ears and neck showed a voluminous formation, with infiltrative character of the parotid gland, neck muscles, and infiltration of the skull base holes with extension to the left jugular hole. The mastoid was clearly invaded (Figures 5, 6).

Subsequently, biopsies were taken from the supraclavicular mass and the bone marrow. The results were compatible with plasmablastic myeloma. In view of the anatomical and pathological findings, chemotherapy and symptomatic treatment were started under the care of the Hematology department. Approximately four months after the initial diagnosis of Bell's Palsy, the patient died at home.

## Discussion

Peripheral facial paralysis can be caused by numerous pathologies, some of them quite rare [6], as in the clinical case presented.

The invasion of the temporal bone by multiple myeloma was first described in 1979 by Lavine et al. [7]. Early diagnosis is challenging since symptoms may develop late in the course of the disease [8]. Plasmablastic myeloma is a morphological subtype of multiple myeloma. It is a rare, monoclonal plasma cell neoplasm with a poor prognosis (average survival of 10 months) [9]. Due to the low incidence of plasmablastic myeloma, there is no consensus on treatment [10].

Considering the frequency of facial nerve paralysis, it is crucial to identify the primary pathology if possible. However, in follow-up appointments, especially in Physical Medicine and Rehabilitation consultations, in addition to focusing on neuromuscular rehabilitation, the physician should conduct a thorough clinical evaluation. Identifying the etiology, even if done during the rehabilitation program, may allow for proper referral and the initiation of treatment as early as possible.

## Conclusions

Bell's palsy is a diagnosis of exclusion. Therefore, if the symptoms do not improve as expected, it is essential to reconsider the differential diagnosis and carry out a complete re-evaluation to rule out neoplasms or other pathologies. Complementary diagnostic tests, particularly CT scans and MRIs, play an important role.

The Physical Medicine and Rehabilitation specialist plays an important role in the whole process, as he or she is often the clinician who has the closest contact with the patient. Lastly, the complexity of this pathology makes interdisciplinary health teams increasingly important.
